# Supplementary material for: Schizophrenia-Mimicking Layers Outperform Conventional Neural Network Layers
Source: Front Neurorobot. 2022 Mar 28;16:851471. doi: 10.3389/fnbot.2022.851471 (PMC8995800; doi:10.3389/fnbot.2022.851471)
Supplement: Supplementary file 1 [file Data_Sheet_1.PDF]

Supplementary Material

Supplementary Materials for:

Schizophrenia-mimicking layers outperform conventional neural network layers

Ryuta Mizutani, Senta Noguchi, Rino Saiga, Yuichi Yamashita, Mitsuhiro Miyashita, Makoto Arai, and Masanari Itokawa

Supplementary Table 1. (A) Configuration of network A. Sz, schizophrenia connection layer. The Number-of-parameters column shows the number of trainable parameters before the parameter reduction.

Layer	Output size	Number of parameters	Options
Input	28 × 28		
Sz	512	401,920	Parameter reduction: 0–95%
Output	10	5,130	

Supplementary Table 1. (B) Configuration of network B. FC>Sz, trained as a fully connected layer and evaluated using a weight window. The Number-of-parameters column shows the number of trainable parameters before the parameter reduction. *Dimensions of these hidden layers were set equal to each other and varied to examine the effect on the connection alteration.

Layer	Output size	Number of parameters	Options
Input	28 × 28		
Fully connected	64–1024*	50,240–803,840	
FC>Sz	64–1024*	4,096–1,048,576	Bias vector was disabled.
Output	10	650–10,250	

Supplementary Table 1. (C) Configuration of network C. Sz, schizophrenia connection layer; Conv, 2-dimensional convolution layer with a kernel size of 3 × 3; FC, fully connected layer. The Number-of-parameters column shows the number of trainable parameters before the parameter reduction.

Layer	Output size	Filter size	Number of parameters	Options
Input	32 × 32 RGB			
Conv	32 × 32 × 32	32	896	Zero padding
Conv	30 × 30 × 32	32	9,248	No padding
Maxpool	15 × 15 × 32			Pooling 2 × 2, dropout: 25%
Conv	15 × 15 × 64	64	18,496	Zero padding
Conv	13 × 13 × 64	64	36,928	No padding
Maxpool	6 × 6 × 64 (= 2304)			Pooling 2 × 2, dropout: 25%
Sz<br>or FC	512		1,180,160	Parameter reduction: 0–90%<br>L_1 regularization
Output	10		5,130	

Supplementary Table 1. (D) Configuration of network D. FC>Sz, trained as a fully connected layer and evaluated using a weight window. Conv, 2-dimensional convolution layer with a kernel size of 3 × 3. The Number-of-parameters column shows the number of trainable parameters before the parameter reduction.

Layer	Output size	Filter size	Number of parameters	Options
Input	32 × 32 RGB			
Conv	32 × 32 × 32	32	896	Zero padding
Conv	30 × 30 × 32	32	9,248	No padding
Maxpool	15 × 15 × 32			Pooling 2 × 2, dropout: 25%
Conv	15 × 15 × 64	64	18,496	Zero padding
Conv	13 × 13 × 64	64	36,928	No padding
Maxpool	6 × 6 × 64 (= 2304)			Pooling 2 × 2, dropout: 25%
FC>Sz	512		1,179,648	Bias vector was disabled.
Output	10		5,130	

Supplementary Table 1. (E) Configuration of network E. Conv, 2-dimensional convolution layer; SzConv, 2-dimensional schizophrenia convolution layer; FC, fully connected layer. A kernel size of 3 × 3 was used for all convolution layers. The Number-of-parameters column shows the number of trainable parameters before the parameter reduction.

Layer	Output size	Filter size	Number of parameters	Options
Input	32 × 32 RGB			
SzConv	32 × 32 × 64	64	1,792	Zero padding, parameter reduction: 0 or 42%
Batch norm.			256	
Conv	32 × 32 × 64	64	36,928	Zero padding
Maxpool	16 × 16 × 64			Pooling 2 × 2, dropout: 25%
Conv	16 × 16 × 128	128	73,856	Zero padding
Batch norm.			512	
Conv	16 × 16 × 128	128	147,584	Zero padding
Maxpool	8 × 8 × 128			Pooling 2 × 2, dropout: 25%
Conv	8 × 8 × 256	256	295,168	Zero padding
Batch norm.			1,024	
Conv	8 × 8 × 256	256	590,080	Zero padding
Batch norm.			1,024	
Conv	8 × 8 × 256	256	590,080	Zero padding
Maxpool	4 × 4 × 256			Pooling 2 × 2
Conv	4 × 4 × 512	512	1,180,160	Zero padding
Batch norm.			2,048	
Conv	4 × 4 × 512	512	2,359,808	Zero padding
Batch norm.			2,048	
Conv	4 × 4 × 512	512	2,359,808	Zero padding
Maxpool	2 × 2 × 512			Pooling 2 × 2
SzConv	2 × 2 × 512	512	2,359,808	Zero padding, parameter reduction: 0 or 60%
Batch norm.			2,048	
SzConv	2 × 2 × 512	512	2,359,808	Zero padding, parameter reduction: 0 or 60%
Batch norm.			2,048	
SzConv	2 × 2 × 512	512	2,359,808	Zero padding, parameter reduction: 0 or 60%
Maxpool	1 × 1 × 512			Pooling 2 × 2
FC or Sz	4096		2,101,248	Parameter reduction: 0% (FC) or 50% (Sz)
FC or Sz	4096		16,781,312	Parameter reduction: 0% (FC) or 50% (Sz)
FC or Sz	1024		4,195,328	Parameter reduction: 0% (FC) or 50% (Sz)
Output	10		10,250	

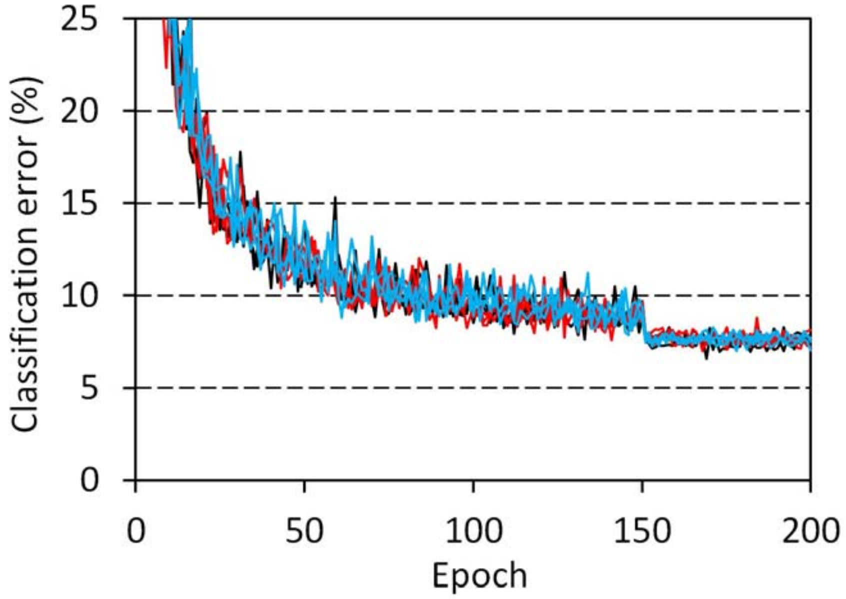

Supplementary Figure 1. Progress of training in the CIFAR-10 classification tasks using the VGG16 network. The three top layers were replaced with schizophrenia connection layers in addition to using schizophrenia convolution layers in the last convolutional block (Table 1, E). Parameter reduction was set to 50% in the schizophrenia connection layers and 60% in the schizophrenia convolution layers. This double-schizophrenia VGG16 network was trained for 3 sessions, and the resultant errors are plotted in cyan. Results for the schizophrenia VGG16 network with a 60% parameter reduction only in the last three convolution layers are drawn in red. Those of a control network without schizophrenia layers are drawn in black.

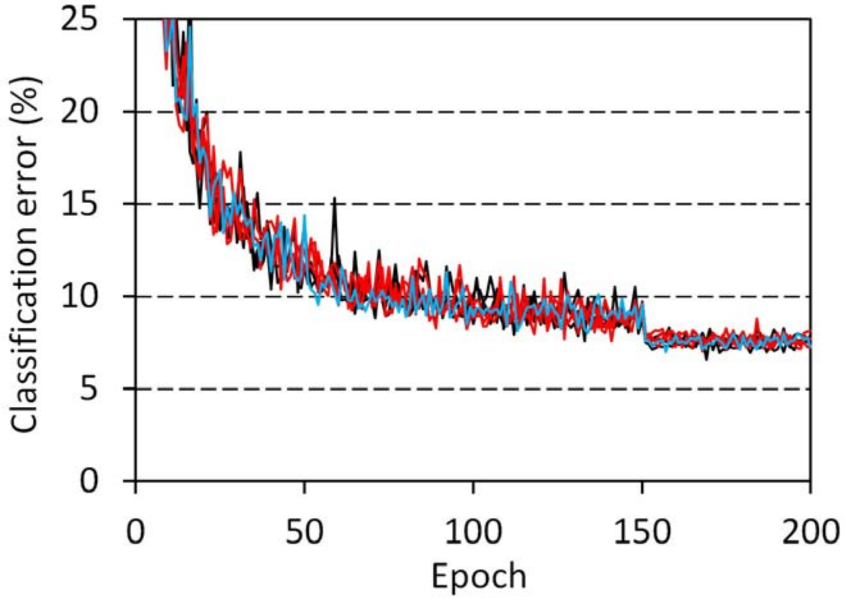

Supplementary Figure 2. Progress of training in the CIFAR-10 classification tasks using the VGG16 network. In order to analyze the responses of the learned filters (Figure 5), the first convolution layer was replaced with a schizophrenia convolution layer in addition to using schizophrenia convolution layers in the last convolutional block (Table 1, E). Parameter reduction was set to 41.7% in the first convolution layer and 60% in the last three convolution layers. This schizophrenia VGG16 network was trained for 1 session, and the resultant errors are plotted in cyan. Results for the schizophrenia VGG16 network with a 60% parameter reduction only in the last three convolution layers are drawn in red. Those of a control network without schizophrenia layers are drawn in black.
